# Socio-cultural change in conflict and post conflict settings: five decades of giving birth in Cambodia

**DOI:** 10.1186/s13031-019-0237-6

**Published:** 2019-11-15

**Authors:** Bandeth Ros, Gillian Lê, Suzanne Fustukian, Barbara McPake

**Affiliations:** 1 ReBUILD Consortium, Melbourne, Australia; 2 Nossal Institute for Global Health, Melbourne, Australia; 3 Queen Margaret University, Musselburgh, UK

**Keywords:** Cambodia, Giving birth, Socio-cultural change, Life histories

## Abstract

**Background:**

This paper explores the changing experience of giving birth in Cambodia over a 53-year period. During this time, Cambodian people experienced armed conflict, extreme privation, foreign invasion, and civil unrest.

**Methods:**

An historical perspective was used to explore the changing place and nature of birth assistance given to Cambodian women between 1950 and 2013. Twenty-four life histories of poor and non-poor Cambodians aged 40–74 were gathered and analysed using a grounded thematic approach.

**Results:**

In the early lives of the respondents, almost all births occurred at home and were assisted by Traditional Birth Attendants. In modern times, towards the end of their lives, the respondents’ grand-children and great grand-children are almost universally born in institutions in which skilled birth attendants are available. Respondents recognise that this is partly due to the availability of modern health care facilities but also describe the process by which attitudes to institutional and homebirth changed over time. Interviews can also chart the increasing awareness of the risks of homebirth, somewhat influenced by the success of health education messages transmitted by public health authorities.

**Conclusions:**

The life histories provide insight into the factors driving the underlying cultural change: a modernising supply side; improving transport and communications infrastructure. In addition, a step-change occurred in the aftermath of the conflict with significant influence of extensive contact with the Vietnamese recognised.

**Trial registration:**

None.

## Introduction

Up to 1 billion people live in fragile settings, with many of them among the world’s poorest and most vulnerable. By 2030, it is estimated that 43–60% of the world’s extreme poor will live in fragile and conflict-affected contexts [1]. Health systems in such contexts are severely affected by conflict and their rebuilding raises numerous challenges. Often the state is unable to supply even basic health care services to a large proportion of the population; may have ineffective or non-existent referral systems; lack infrastructure to deliver health care services; and have weak or non-existent managerial and policy making capacity. During and after conflict, many new non-state actors get involved in a health care system including international organizations; Non-Governmental Organizations (NGOs); and private entrepreneurs at a time when the state often cannot manage such plurality, and capacity to exert authority is low. At the same time, some of the most important decisions on health system rebuilding are made in the post conflict “moment” [2] but it is rare that aid agencies take a longitudinal view on the results of those decisions [3, 4].

**Fig. 1:**
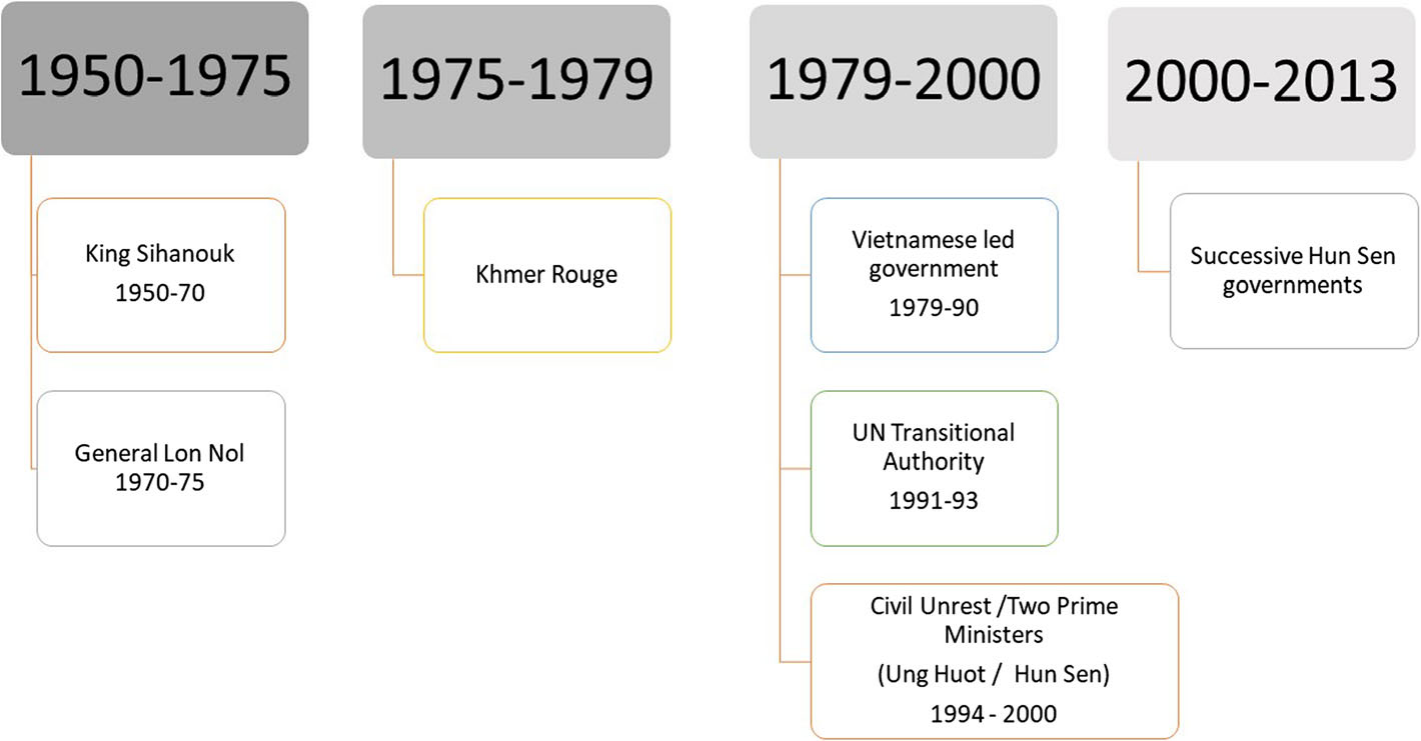
Brief Overview of Political Regimes

The longevity of conflict in Cambodia can act as a case-study on health system rebuilding over a longer term than is normally considered. During the period of research for this paper, political regimes changed eight times. The regimes mentioned in this paper set out below in a timeline, for clarity (see Fig. 1).

Often such a story is told top-down in terms of government policy and international aid decisions. We offer an alternative view of history; on how service supply was impacted by conflict and how families and households responded. This is an important perspective to understand since the functioning of a health system is an interaction between the supply of services and the population’s response. Factors shaping the latter are rarely the focus.

In Cambodia, the pre-Khmer Rouge conflict; the years of genocide during the Khmer Rouge regime; and the post-Khmer Rouge occupation by Vietnamese forces severely compromised the development of the health system. Despite early attempts to modernize the health-system following independence, the state’s capacity to meet the health needs and demands of the population were largely unmet until after the Paris Peace Accords in 1991, some 12 years after the end of the infamous Khmer Rouge regime. From 1992, the state of Cambodia began to rebuild its health system, constructing health infrastructure and introducing professionally trained health personnel and practices. However, relatively little is known about what shaped the population’s response over the transition from decades of absent or limited care for maternal health to a pluralistic system comprising modern health care methods and facilities provided through public and private health care services as well as the continuing presence of Traditional Birth Attendants (TBAs) and other traditional practitioners (see [5]).

In this paper, we examine the responses by households to diverse formal and informal service options for giving birth in Cambodia over a 53-year period. Decision-making about seeking care for giving birth was chosen as a probe for the study since most households will repeatedly experience births from one generation to the next and make choices about how and where to support them. The research questions to which we respond in this paper are as follows:

- How have care seeking behaviours for the life event of pregnancy and birth evolved over the period 1950 to 2013?
- What have been the major influences on this evolution?
- What role did the protracted conflict play?

By way of context for these questions, we consider a brief history of birth assistance services in the country.

### A history of service supply: birth assistance from the colonial period to twenty-first century

In healthcare facilities managed under the French Protectorate (1867–1946) such colonial health service facilities and charitable organizations, doctors handled care for women giving birth. However, the concept of medical assistance while giving birth was unpopular among the general Khmer population [6].

In 1907, a private maternity clinic in the Cambodian quarter of Phnom Penh was established, targeting the Khmer population, funded by donations from both European and Cambodian notables. By 1911, it was noted that, while the number of births at the facility had doubled from 115 in 1908, the main users were Vietnamese. The rising number of Vietnamese clients could be explained by their greater familiarity with European practice of maternal health care [6].

Although the number of facility-based Khmer births had risen by 1926, hospitals were still seen as places of last resort by most Khmer, to be used by those who were abandoned by their husband or family or had unexpected labor during travelling. Services remained unattractive, possibly due to the nationality of doctors and midwives (French of Vietnamese), and cost (ibid).

During Prince Norodom Sihanouk’s administration (1950–70), a medical modernization program was initiated. By 1963, Cambodia had about 140 trained midwives with a three-year education delivered by the Ecole Royale d’Infirmieres et de Sages Femmes d’Etat, established in 1950 [7]. At the same time, about 400 TBAs were given short training courses of 6 months. The total number of childbirths in 1963 was estimated at 236,000, of which midwives and TBAs assisted a total of 55,000. Overwhelmingly, birth still took place at home (ibid).

Childbirth at the few public facilities that existed declined during the Khmer Rouge period (1975–79), and the training institution, Ecole Royale d’Infirmieres et de Sages Femmes d’Etat, was closed. A few major hospitals in Phnom Penh and in the provinces still functioned during this time; however, such hospitals (with trained medical staff) and modern pharmaceuticals were reserved for the highest Khmer Rouge leadership, loyal cadres, and the most loyal residents of the countryside. Even TBA services were usually accessed by these cadres. Pregnant women and the general population were taken care of by *pet padevat* (healthcare workers) using medicines made from locally available plants. Since *pet padevat* had almost no technical skills and were committed to the Khmer Rouge ideology, women received little empathy and very poor-quality treatment and care (ibid.). The birth rate declined from 43 per 1000 in April 1975 to about 28 per 1000 in late 1978 for those who were rural dwellers before the regime. For new or existing urban dwellers before the regime, birth rates declined faster, from 32 per 1000 in April 1975 to around 10 per 1000 by late 1978 [8]. Nationally, the middle range estimate for birth rate was between 20 and 25 per 1000 during 1976–1978 [9].

With a change in political regime, the Ecole Royale d’Infirmiere reopened in 1980 and was renamed the Technical School for Medical Care [7] at the same time, four Regional Training Centres serving Battambang, Stung Treng, Kompot, and Kampong Cham provinces were established by the Ministry of Health to train paramedics including primary and secondary midwives following a standard curriculum [10] Even with this effort, a joint Cambodian Ministry of Health and World health Organization (1993) report estimated that the maternal mortality rate remained high at 900 per 100,000 live births, but which was likely higher since home births were not accounted for [11].

During the period of the United Nations Transitional Authority in Cambodia (UNTAC) (1991–1993), and through the various Hun Sen led governments, Cambodia received substantial financial and technical development aid. Health has always been a focus; maternal and child health (MCH) was placed at the center of most aid interventions following global initiatives of the time, such as the Safe Motherhood Initiative of 1987, the 1994 International Conference for Population and Development (ICPD) and the 2000 United Nations Millennium Development Goals (MDGs). However, despite the early commitment to midwifery training shown by the Ministry of Health, the government discontinued midwifery training between 1996 and 2002, resulting in a severe shortage of midwives [7, 10].

The state’s Health Sector Strategic Plans (HSSP) - HSSP1 (2003–2007) [12] and HSSP2 (2008–15) have since sought to reduce maternal, new-born and child morbidity and mortality [13]. From 2002, efforts to increase the education and supply of trained midwives, both primary and secondary, intensified [10]. Emphasis was given to training primary midwives and was initially conducted at the provincial level, based on 3 years of nursing followed by 1 year of midwifery training (3 + 1). Responsibility for training returned to the Technical School for Medical Care and the four Regional Training Centres where a Diploma was initially offered, followed by a 3 + 1 degree, and, in 2012, a 4 year direct entry Bachelor degree in midwifery was established [7]. This brief history provides a context to understand how families and households sought assistance in giving birth across different political regimes. In the article that follows, we first detail our method before considering the interview results. Excerpts from interview can be seen as changes in demand over the last 53 years. We conclude with our thoughts on the effectiveness of health system strengthening in conflict and post-conflict periods.

## Method

The data for this paper were generated through a larger study focused on health-seeking behaviour and spending faced by Cambodians across different political regimes from 1960 onwards. The study was conducted by the ReBuild Research Consortium from April to September 2013, in which a life history approach was used. Life histories can shed light on shifts in attitudes and behaviours and the reasoning behind such shifts [14]. They enable a long-term perspective on societal change and how individuals were affected by such change.

Twenty-four life histories were collected on episodes of illness, deaths and births; in this paper, the interview data focused on giving birth were extracted. The interview guides covered the following topics:

- When was the birth and who was giving birth?
- What did you do?
- Why did you do that?
- What led you to decide where to go?

The interview guides were developed based on the research questions given earlier (in short, the evolution of care seeking for birth assistance over the period chosen; and the major influences on this evolution including the role of protracted conflict). The field researcher pretested the interview guides in a village in Takeo province and adjust preliminary questions through a discussion with her research team.

The study was carried out in four Operational Health Districts (ODs) of Phnom Penh and two in Takeo province. These ODs were selected from the locations in which health NGO partners in the larger ReBUILD project were working at the time of the study.

A total of 24 interviewees were purposively recruited using the following criteria: levels of poverty, age, and continuing residence in Cambodia. First, 11 poor and 13 non-poor people were interviewed. Poor households were identified through discussion with village chiefs, local health NGO operators and cross-checking to a list of persons who were already identified as poor by the national Identification of Poor Households, a government programme in Cambodia (known as ID Poor in short). The national ID Poor government program followed seven steps, one of which was household scoring based on a questionnaire with a set of proxy indicators for poverty [15]. For respondents that we classified as non-poor, we selected persons who were not included in the ID Poor program, through discussion with the same stakeholders noted above. To ensure that respondents had sufficient memory recall of major birth events experienced by the whole household, and significant historical perspective including the period of interest, only heads of households, male or female, who were 40 years old or older were recruited. Ideally, we would have restricted respondents to those at least born by the beginning of the period in which we are interested, but it proved difficult to find enough persons aged 63 or more who were unaffected by medical conditions affecting memory.

Ethics approval was received from both the National Ethics Committee for Health Research in Cambodia and Queen Margaret University in the UK. Because most participants in this study were illiterate and were reluctant to provide written consent, explicit verbal consent following an explanation of the study and its objectives was obtained by the researcher. The researcher had stated to all participants that there was little risk involved in this study; that their participation was voluntary; and they could withdraw their participation at any time.

Each interview lasted between 2.5–4.5 h. The field researcher positioned herself as a guide and facilitator and allowed time for respondents to recall events. Probing questions were used to guide the respondent in that recall. Interviews were audio-recorded and transcribed first in Khmer language from the audio record and then translated into English. At the time of the interview, the householders were living in urban or rural settings, but place of residence changed many times over their lifetime and the rural/urban distinction proved unhelpful to categorizing respondents’ experiences. A coding framework was developed structured in chronological order as follows; birth event; type of service sought; location of service; when sought; cost. Transcripts were reviewed, and excerpts inserted into the framework. The codes were compared across time and themes. Reflections were shared between co-authors through several stages to confirm, challenge, and feedback into further refinement of the themes presented here.

We recognise four potential limitations of our analysis. The data was obtained from two out of 25 provinces and could therefore reflect a regional perspective although the high mobility of the respondents between provinces and rural and urban areas suggests this is unlikely. Further, since the data source was interviews, it is subject to limitations of interviews generally, due to reliance on memory recall. Also, the status of ID poor and non-poor participants in this study reflects their financial status in 2013 not their historical status in other periods since poverty levels changed over their life course. Last, the data used in this study were based on research carried out in 2013 (tied to the larger ReBUILD project). Further research is needed to complement the findings presented here on giving birth from 2014 onwards.

We now turn to the interview results.

## Results

**Fig. 2:**
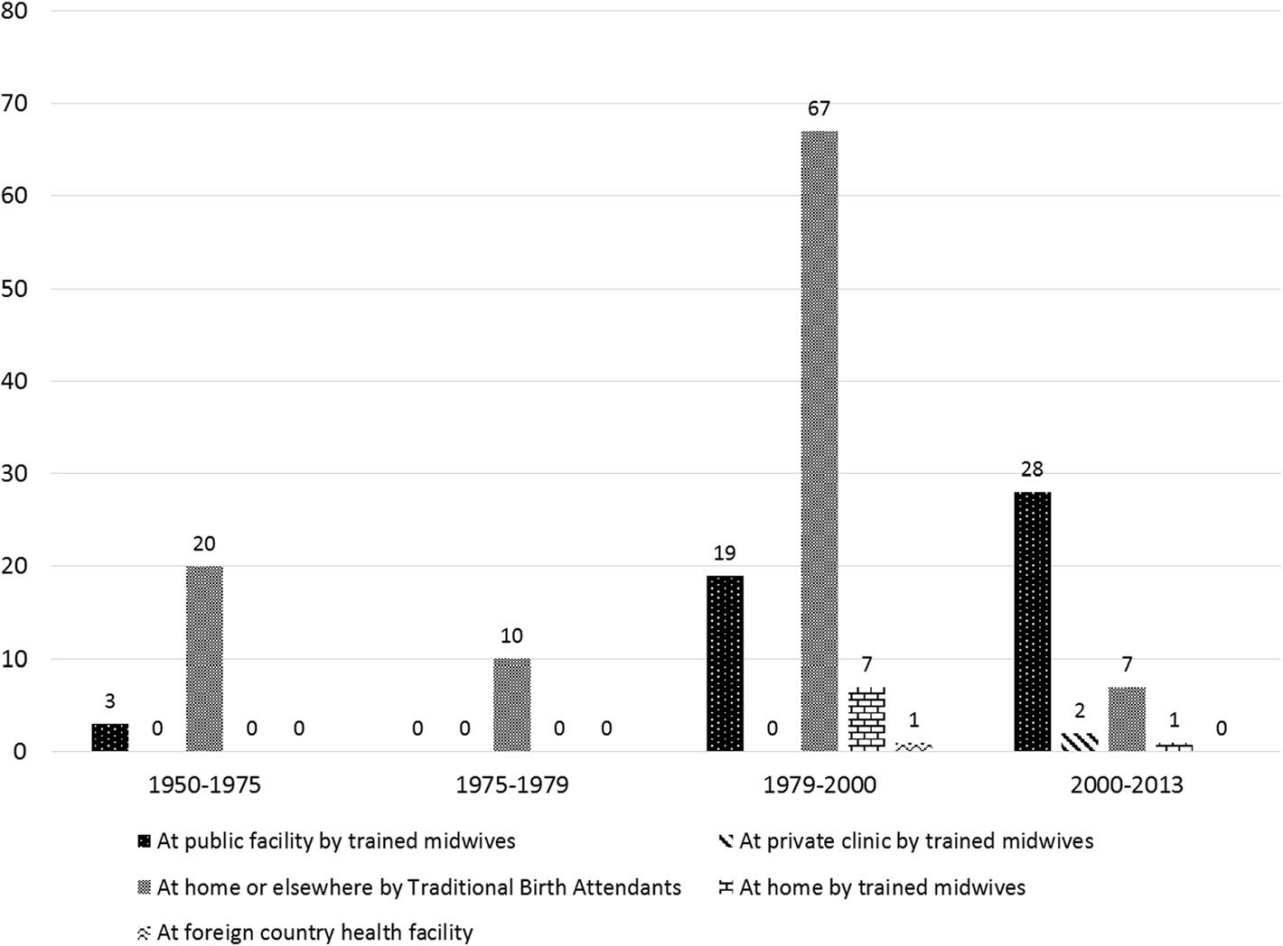
Number of Birthing Experiences by Types of Assistance Reported

Five forms of giving birth were reported by interviewees, based on 165 experiences over the last 53 years (see Fig. 2). We consider how care seeking for pregnancy and birth has shifted during each period, starting with the earliest in our study.

### 1960–1975

During this period, TBAs were popular; in rural areas where public facilities were rare as well as in urban areas, where public facilities were available. TBAs could be persons in a woman’s village or in neighbouring villages; family members or not; but those with extensive experience in assisting birth. The ‘TBA’ may not necessarily persons be clearly designated as such. The perceived difficulties associated with giving birth (the Khmer term for birth delivery, ‘*ChhlongTonle’*, means ‘crossing the river’) meant that women relied on TBAs who were recommended by their family, friends or fellow villagers whose capacity to assist was known and trusted. A woman may use the same TBAs at every birth, if that TBA was available. The most frequent reason given for TBA use was that TBA was considered safe and that it was a common practice. Another, less common explanation was inaccessibility to public facilities due to a combination of lack of facilities, distance, and lack of transportation. After giving birth, women were often recommended to take traditional medicine and follow traditional practices.

“Traditional birth attendants were very common and preferable. People spread the word, from mouth to mouth, about how to stay safe during pregnancy and in labour, especially about selecting Khmer herbal medicine and the practice of getting warm through fire for two or three months [after giving birth]” (M, 56, Take_24).

“Pregnant women were rarely brought to hospital to deliver the baby because it was too far, and they may give birth on the way. Another choice was to carry them with a hammock. There was nothing else” (M, 74, Phnom Penh_7).

“People in the past did not really believe in medical healthcare for giving birth; they believed in the TBA … [she] wrapped threads around the umbilical cord of the baby very tightly … and then cut them off using tailor’s scissors” (M, 74, Phnom Penh_7).

Interviews showed that even when respondents lived close to a hospital, they still used TBAs when they perceived that giving birth would be safe, with no apparent complications (e.g. bleeding or prolonged labour pain).

“Q: When you had your fourth child, you were in Kampong Cham Town and you lived near the hospital - why didn’t you bring your wife there?

A: There was a TBA living near my house, and my wife had no difficulty in giving birth” (M, 74, Phnom Penh).

One maternal death was reported by one respondent in this period, believed by the respondent to have been caused by a spirit.

“My mother died after delivering a baby … I was there when she died. Both she and her the baby died on the same day … . After giving birth, a woman is supposed to sleep on a warm bed above a stove with fire heating, and we have to stick a nail on the bed otherwise “*arb*” (a kind of demon) would come to disturb the woman. We can’t see that demon, but the mother of the new-born can see it. Then, she would open her eyes widely and her body temperature would increase. She could die if no one can help” (F, 45, Phnom Penh_8).

New-born deaths were mentioned by three respondents. Deaths were often associated with illness of the new-born or was perceived to be caused by a spirit.

“Q: When did your first child die? A: She died when she was a half month old. She had oral thrush and she refused breast. Sometimes she drank my breast milk, but often vomited. After a period of time, she died” (F, 63, Takeo_6).

### 1975–1979

During the Khmer Rouge regime, none of the interviewees reported giving birth at health facilities or hospitals. As noted earlier, where a small number of health facilities had survived, they were reserved for elite, revolutionary, categories. Even access to TBAs became difficult because TBAs were often assigned to work in different locations. In addition, due to forced labour– a common experience during the Khmer Rouge period - the interviewees moved often between work locations and were expected to work as normal, usually on a punishing schedule until the day of giving birth.

“When we reached Prey Veng, my mum couldn’t walk any longer. She was pregnant, and her legs were swollen. So, we went into the village to rest there. We had planned to go to our hometown, but Pol Pot soldiers sent us in a different direction” (F, 47, PP_12).

“I was moved [to a new place] three days before I gave birth to my fifth child. My husband went to [the TBA] for help when I was about to give birth. I looked after about ten kids when I was moved to that region. I was assigned to babysit those kids because I had just given birth and, thus, they let me do easier task. After two months, I returned to work, planting seedlings and rice harvesting” (F, 63, Takeo_13).

For one woman, there was no TBA, only the elderly women in her work group.

“Even though I was getting morning sickness, I was forced to carry soil and dig canals … I did this work until I was pregnant for five months and then I was transferred to transplant rice seedlings [and after that] to husk the rice and then carry it to the kitchen. I worked until I couldn’t anymore and then I was allowed to rest at home until giving birth. I was crying while working because my stomach was getting pain when I worked too hard and I was trying to massage it to reduce the pain and then continued to work. Somehow, I didn’t dare to complain about this work because if it was heard I would be imprisoned and still forced to work as well … there wasn’t much Khmer medicine at that time and the elders told me to boil a kind of herb to ease labour … if smoke had been seen from our site, they would have come to check it so I hid the herb boiling from them. One elder boiled the herbs and the other stayed outside in case someone came to check the place. If we saw someone walking to us from the distance, we would have smothered the fire” (F, 53, PP_11).

The same woman mentioned the new-born death of her first child during this period.

**“**Q: When did you have the first child? A: After being married for 1 year, I had a daughter. This child died a week after delivery. At that time, before delivery, I was transplanting at the rice field. After I came out of the field, I went into labor and so I went to lie in bed to rest, but then I gave birth immediately” (F, 53, PP_11).

### 1979–2000

During this period, new patterns of maternity services started to emerge, including homebirth with trained midwives; and giving birth at public facilities. Interviewees were still highly dependent on TBAs after the fall of the Khmer Rouge in 1979 through to the late 1990s, not least because they were available and close-by to offer immediate assistance.

“At that time, there was still no hospital [in Angroka]. However, TBAs were available in the village since the Pol Pot regime had collapsed, and so people who were traditional birth attendants returned to live in the village” (F, 53, Phnom Penh_11).

“It was because there was no hospital here at that time. A hospital was available at the district when my wife had the third or the fourth children but even if a hospital/medical centre [was available] midwives were not … so people always delivered at home with TBA” (M, 54, Takeo_14).

“All my seven children were delivered by traditional midwives. From 1979-1983 there were no doctors [and no] proper doctors from 1979-1990. There were some physicians in the commune, but not enough medicines were available” (M, 67, Takeo_21).

The civil war between the remnants of the Khmer Rouge and the government troops continued until 1998 in Battambang province where one interviewee lived at that time. Landmines along the way to the district town also reduced the accessibility of public hospitals as reported by the interviewee.

“There were hospitals only in [the] district towns but not … . where I was living. During that time there were still some remnants of Khmer Rouge … those remnants didn’t hurt civilians because they were afraid of the government’s troops. Sometimes they came to ask for rice … Because the distance was far, we were worried that I might deliver the baby on the way, and if it was so, we couldn’t find any TBA on time... there were landmines along the way as well. So, I tried to labour at home, but I didn’t know what time I delivered my child because I was unconscious” (F, 55, Takeo_15).

Another interviewee talked about the deployment of Vietnamese troops near the Cambodian-Vietnamese border (Takeo province) where the remnants of Khmer Rouge remained. She dared not travel outside the village to access public facilities, for safety reasons.

“Vietnamese troops lived there. However, people were afraid of [them] … we didn’t dare to go out at night. During the day, we needed a few people to accompany us or Vietnamese troops could harm us” (F, 63, Takeo_18).

The presence of trained Vietnamese midwives, medical workers or neighbours in 1980s to support childbirth and postnatal care of Cambodian women was also reported. One interviewee witnessed Vietnamese midwives providing services at hospital in 1980. She also recalled receiving advice on giving birth at hospital from a Vietnamese neighbour. In 1989, the same interviewee received support from a Vietnamese midwife to deliver her fifth child at home. Another interviewee recollected receiving postnatal care from Vietnamese medical providers in 1984.

“My neighbours, some elderly people, and the Vietnamese that lived near me advised me to go to hospital. They said “you should go to hospital; Vietnamese people never use TBA because they were afraid of risks of TBA. I listened to them, so I went to hospital and I could deliver baby safely. It’s near our house … when I was in labour, I rushed to hospital where they provided 24 hours service. All the medical staff were Vietnamese, and we had very few Khmer medics from the brutal regime” (F, 60, Phnom Penh_2).

“I did not know any hospital at that time. I only recalled that it was flooding season. We did not have money, so I was delivered the baby by a Vietnamese midwife” (F, 60, Phnom Penh_2).

“[I] delivered my son at the hospital. But I didn’t have a medical midwife to help me. [I] used only Khmer traditional midwife … Vietnamese physicians were busy treating injured people … After the birth, [I used] herbal medicines plus the help from the Vietnamese medical staff. They gave me some injections.” (F, 52, Phnom Penh_1)

Two new-born deaths and one stillbirth were reported by respondents in the early 1990s. The reasons were illness of the new-born and heavy work done by women during pregnancy.

By the late 1990s, hospitals were established at district levels in Kompong Cham and Takeo provinces where interviewees were living at the time. However, services were not yet available at community level. It was challenging for those who lived far from district towns to access public services. The combination of the cost of giving birth at a hospital, generalized household poverty at this time, along with a distrust of health worker motivations, also influenced decision-making.

“There was a referral hospital at district as well as one just established about 2-3 km from home. But I didn’t have a motor bike … at that time we didn’t have a health centre in the community” (F, 41, Phnom Penh_6).

“Sometimes I thought of going to hospital, but I had no money … I witnessed some doctors not taking care of patients if they didn’t have money. Those doctors did not even look at the face of those poor patients. However, if patients had money, they were helped quickly. My sister experienced that” (F, 41, Phnom Penh_6).

Whilst TBAs continued to be popular, hiring private trained midwives to perform deliveries at home also emerged as a common behaviour during this period. One interviewee talked of hiring a former midwife (who had survived the Khmer Rouge regime) to assist his wife at home; four others recalled hiring newly-trained midwives during the late 1990s. The hiring of private midwives came after people became more aware of the risks of giving birth and availability of the service, and so they demanded reliable services as they faced inaccessibility to public facilities.

“We became aware of who, in former times, were midwives and asked them for assistance for giving birth … (they also had) their own equipment to assist us [ … ] unlike the TBAs” (M, 53, Takeo_14).

**“**Q: You used the TBA so why did you invite the midwife? A: It was because it was a very long birth. I didn’t feel the pain and I didn’t really know when to push. After one baby was born, then they said that there was another baby coming. I lost my energy, so my husband went to invite a private midwife” (F, 46, Phnom  Penh_11).

“Q: You chose to deliver the baby at home with the assistance of a trained midwife, but not a TBA? Why? A: There were some TBAs still, but most of them were getting old. Trained midwives started to be available at that time and people began to rely on them for giving birth. With the experience of my other sisters’ who had given birth, my mother suggested to ask a trained midwife to help me during birth. The medical midwife also had her own equipment such as scissors and knives” (F, 55, Takeo_03).

Border residents reported seeking care in neighbouring countries for a complicated case, when their income allowed. Clearly, this also relied on improved roads and availability of transportation. One Takeo resident lived close to Vietnam and discussed giving birth to her 11th child.

“Before I went to Vietnam, I saw a neighbour who died after she delivered a baby at home and was bleeding. After I saw that, I wanted to go to hospital, and did not dare to deliver it at home … A village physician suggested I go to Vietnam and another physician knew Vietnamese, so he accompanied me” (F, 57, Takeo_20).

An increase in public facility use in this period can also be observed from interviewees, especially among urban residents. Transportation also improved faster in urban areas and interviewees appeared more aware of risks in giving birth, gained through daily conversations with neighbours and friends. These accounts swayed five interviewees to deliver at a hospital.

**“**Q: How was your third child [delivered]? A: In hospital. At that time, the transportation has improved a lot” (M, 74, Phnom Penh_7).

“Where was your second child born? A: The second child was born at the Red Cross Hospital near the Independent Monument … I started having prenatal check-up with my second child … When I moved here where I'm closer to medical staff, I heard them talking about pregnancy care. Then, I became worried, so I just went there to have prenatal test because it's also very close to my place. My elderly neighbour also advised me to have prenatal test” (F, 46, Phnom Penh_9).

In the early post-conflict period, a family of military personnel could also gain free access to care in urban areas. For example, the interviewee below recalls how she gained free care through her nephew:

“[He] told me during my pregnancy that when I was in labour, I should inform him, and he would take me to military hospital so that I did not need to pay. I was even given food, scarf and towel. When I was in labour, he took me to the hospital by motorcar at midnight. I rested there for three days … I had my name as a wife of my nephew. He had a soldier certificate” (F, 52, Phnom Penh_1).

### 2000–2013

During this period, interviewees reported four types of maternity services: homebirth with TBAs; homebirth with trained midwives; giving birth at a public facility and an emerging use of trained midwives at private clinics. Use of private clinics depended on slowly improving economic conditions for the household, the availability of private facilities, and a desire for better quality of service. Use, however, depended on household finances:

“My brother suggested that I go [to a private clinic]. He said … he would pay for me. I didn’t know why he suggested this clinic, but all five children of my brother were delivered there. His workplace bought him an insurance there. My brother works for the International Red Cross” (F, 47, Phnom Penh_12).

The interviews also revealed a declining reliance on TBAs that was linked to ability to pay for formal providers: interviewees, and the generations that followed them, who could afford the cost switched to public facilities. Those who were unable to pay continued to rely on TBAs.

“Most families would go to hospital now. However, there are still some families – one among 100 families, who are too poor to afford to go to hospital – still use services from TBAs. However, my family has money, so we go to hospital … the hospital asked us first if we had money or not” (M, 57, Takeo_24).

“I did not have money to go to hospital... one bed cost 40,000KHR (USD10.46)^1^ and we need to spend on eating as well. The hospital gave food to us, but we could not eat it” (F, 44, Takeo_22).

Public hospitals became a service of choice for critical conditions or where complications in giving birth were anticipated. In this period, reference was also made to social protection schemes such as the Health Equity Fund for the poor, and Community Based Health Insurance.

“The delivery of the second baby was smooth. She [my daughter] only went to the health centre [and] … didn’t spend any money for the maternity service because her family also shared the ID poor card with me” (F, 52, Phnom Penh_1).

Other interviewees noted that, even with the ID poor card, they could not access hospital care due to continued high level of household poverty and health worker attitudes and behaviours.

“Although the doctor did not ask money from me, I did not have money to buy food ... I did not have any means to travel too. I literally had nothing … [the midwives would want] 30,000KHR (USD7.38)^2^ and fruits just like I told you previously. They asked for the money because they touch our blood^3^ and help us deliver the baby. It's normal. I saw the example of my neighbour. She went to a health centre, but [the young midwife] asked her for 30,000 riels (USD7.38), two kilos of fruits and two cans of fruit juice. They said it's because they touched her blood. Those midwives are young, around 17 or 18 years old. They said they did not ask for other prices, but they only asked money for touching my blood. If I did not have money, I could not go” (F, 40, Takeo_17).

“For me, I don’t want to go to hospital. I was also ashamed and shy of nurses and doctors. Health workers had bad words, so I didn’t want to go to hospital. Although I met a good doctor when I gave birth to my previous son, I was afraid that next doctors could be bad and blame me. When they blamed me, I was angry” (F, 41, Takeo_23).

Younger interviewees showed greater pregnancy awareness and changing perceptions of the safest way to give birth, in part due to information and outreach programmes by the government, NGOs, media, community groups, and local authorities.

“Now we have media to raise awareness of people about pregnancy, that women should go to health centre for check-up when expecting a baby, or when they miss their period. The health care system before was far different from now … Now they advertise everything [ … ] and I am a village representative for the health network [ … ] I walk door to door and inform people [ … ] or we invite 10 people for a short meeting. We have 10 packs of washing powder to give to those 10 interviewees and 5000KHR (USD1.25)^4^ for each person, and so people like to participate. When they listen, they hear about pregnancy testing [as well as recognition of infectious diseases … In the past, people didn’t understand about their health, not like now. Now we understand a lot. (F, 52, Phnom Penh_1).

“Now young people prefer to deliver their baby at hospital and there are no TBAs. Giving birth in the hospital is safe because there are a lot of medicines and equipment. I know about this because I went to hospital to take care of my relatives and I observed it. I heard doctors talk about that to the patients. The doctor asked her to return home after staying in the hospital for 24 hours and the midwife came to our house to give her injections. The midwife didn’t allow her to take Khmer traditional medicines, so she drank only warm water. Now people trust trained midwives because they have enough equipment and medicine to help. In the previous time, people trusted TBAs. People’s belief and perception on health care have changed according to circumstances” (F, 55, Takeo_15).

## Discussion

Reduction in maternal mortality over the twenty-first century has been one of Cambodia’s success stories. The Maternal Mortality Ratio (MRR) dropped from 1020 per 100,000 live births in 1990 to 161 by 2015 [16]. One reason for this success is due to the consistent prioritization of maternal and child health in government policy. Other reasons are highlighted in the life histories and include greater awareness of health issues, in part due to better health information and outreach by government programmes; increased access to modern public and private facilities; improved transport and communication; and social health protection in the form of the Health Equity Fund.

The interview results highlight the value of a life history approach, giving rich insights into how socio-cultural change takes place. They chart how attitudes to childbirth of individuals and community have changed, together with changed availability of options for assistance during birth; and how a maternal healthcare system has developed in the country over the last five decades, in tandem with economic security and infrastructure changes, during conflict and post-conflict reconstruction. The life histories highlight how change takes place, and show that change can be rapid, even within a generation (for instance, see interview excerpt Takeo_15 above).

Through the majority of the regimes covered, from King Sihanouk to near present day, TBAs were the main birth attendant along with family members [6]. This is borne out clearly in the interviews, with TBAs preferred even when hospital alternatives were present and nearby. Notably, childbirth had not been problematized as risky, as had happened during the same period in the West. Although a modernization program had been started during Sihanouk’s regime, this was interrupted by ever-increasing civil war and lack of security in daily living.

The horrors of the Khmer Rouge era have been documented elsewhere and are not the subject of this paper. The interview excerpts above convey starkly how isolated the people were during these times of mass genocide, displacement of families, forced labour, starvation, and malnutrition. Our interviews suggest that women giving birth in this era survived because of TBAs and each other. Also, with the extreme violence in the country, and overwhelming destruction of healthcare facilities, the risks of childbirth were simply an added complication to the many other risks women faced daily.

The Vietnamese invasion of Cambodia in 1979 ended direct Khmer Rouge control, though civil unrest in the countryside continued for about 20 years [17, 18]. After 1979, the presence of Vietnamese midwives and healthcare workers to support birth and related postnatal care can be understood as relief support during the immediate post-conflict rebuilding of the health system. While useful, such support was temporary, ad hoc, small scale and available only in urban areas with limited information shared with local people. However, the presence of Vietnamese people and their influence over the culture of childbirth is clear from the interviews and perhaps as important as the growing availability of institutional care under the Vietnamese sponsored government.

The UNTAC period instituted a wider rebuilding of the public health care system with TBAs still the main source of birth assistance over both regimes. However, with exposure to international attitudes, a change in Cambodian’s perceptions of TBAs is seen – TBAs are now thought limited in their scope and quality. This was accompanied by a shift in the perception of risk in childbirth and increased use of medicalized/formalized facilities in both the public sector and a nascent private sector suggesting that the post conflict periods can indeed be fertile opportunities for cultural change. Clearly, a step change in these attitudes occurred in the immediate post conflict period and is attributed to the influence of Vietnamese actors, both friends and neighbours; and authorities of the interim (Vietnamese) government.

The proliferation of public health services accelerated from the year 2000, with significantly increased provision and use of public and private facilities. The commitment shown in the HSSPs to maternal care and the growing capacity and network of facilities indicates a return of the state and social contract with the population. The rising incomes of ordinary Cambodians can be observed along with improved transportation, enabling use of such facilities. By 2014, 89% of Cambodian births were supported by skilled birth attendants [19]. However, there is a divide in service uptake, with rural women and poorer women more likely to use TBAs for birth and post-natal care, for what seem to be economic reasons. In parallel with supply-side change, Cambodians’ views on the safety of home-based care with Traditional Birth Attendants (TBAs) is transforming from being a trusted source of assistance to one seen as risky and outdated.

## Conclusion

In this paper we have explored the changing experience of giving birth in Cambodia over a 53-year period, a time of extensive armed conflict, foreign invasion and civil unrest. We used life histories to trace changing responses to service supply during this period. They provided insight into some of the factors driving the underlying cultural change: a modernising supply side; improving transport and communications infrastructure. In addition, a step-change occurred in the aftermath of the conflict with significant influence of extensive contact with the Vietnamese recognised. In terms of health system strengthening, the interviews bear out the proposition that conflict and post-conflict periods can be periods where socio-cultural change can take place rapidly.

## Data Availability

The datasets generated and/or analysed during the current study are not publicly available due to interviewees not having granted permission for the sharing of their transcripts.

## Footnotes

1 The calculation was based on the USD/KHR historical data available at https://www.investing.com/currencies/usd-khr-historical-data(accessed 20.8.19), the average exchange rate of KHR 3887 for 1USD in 2000 when the birth event occurred.

2 The calculation was based on the USD/KHR historical data available at https://www.investing.com/currencies/usd-khr-historical-data (accessed 20.8.19) at average exchange rate of KHR4,063 for 1USD in 2011 when the birth event occurred.

3 The midwife is in contact with a woman’s blood when assisting a birth - being in contact with blood is perceived as dangerous and those who dare to do so, should be respected.

4 The calculation was based on the USD/KHR historical data available at https://www.investing.com/currencies/usd-khr-historical-data at average exchange rate of KHR4,084 for 1USD in 2013 (accessed 20.8.19).
